# *Actinobacillus pleuropneumoniae* – today's perspective on advances and challenges (2017- 2026)

**DOI:** 10.1186/s12917-026-05791-3

**Published:** 2026-08-11

**Authors:** Isabel Hennig-Pauka, Katrin Strutzberg-Minder, Paul R. Langford

**Affiliations:** 1 Field Station for Epidemiology, University of Veterinary Medicine Hannover, Foundation, Bakum, Germany; 2 Innovative Veterinary Diagnostics (IVD) GmbH, Seelze, Germany; 3 Section of Paediatric Infectious Disease, Imperial College London, South Kensington Campus, London, UK

**Keywords:** Antimicrobial resistance, Biofilm, Diagnostics, Infection models, Immunopathology, Prevention, Swine, Treatment, Virulence

## Abstract

*Actinobacillus pleuropneumoniae* (APP) remains one of the most significant bacterial respiratory pathogens in swine production worldwide, causing porcine pleuropneumonia with substantial economic impact. Although recent reviews have summarized aspects of APP epidemiology, serotyping, surface-associated virulence determinants, and vaccine development strategies, several complementary research areas have advanced rapidly since the last *DISease CONtrol TOOLS* (DISCONTOOLS) 2017 report on this bacterium. These include antimicrobial resistance surveillance, chronic persistence mechanisms such as biofilm formation, refined experimental infection models, intracellular survival within host immune cells, and the immunopathological processes driving lung injury. This review focuses on these practical and mechanistic dimensions, emphasizing their relevance for herd-level control, outbreak management, and future therapeutic approaches. Recent genomic studies of multidrug-resistant strains, as well as the development of improved molecular diagnostic tools, are also discussed. Collectively, emerging evidence suggests that APP should not be viewed solely as an extracellular pathogen causing acute disease outbreaks, but rather as an adaptable organism capable of persistence, immune modulation, and endemic circulation in modern pig populations. Addressing these challenges will require integrated strategies that combine antimicrobial stewardship, improved surveillance, and a deeper understanding of host–pathogen interactions.

**Supplementary Information:**

The online version contains supplementary material available at 10.1186/s12917-026-05791-3.

## Background

Porcine pleuropneumonia, caused by *Actinobacillus pleuropneumoniae* (APP), continues to represent a major challenge for the global swine industry. For this reason, APP was still selected as one of the priority livestock diseases in the *DISease CONtrol TOOLS* (DISCONTOOLS) database in 2024 [1]. A DISCONTOOLS disease and gap analysis was undertaken by an expert group of veterinarians, microbiologists, epidemiologists and industry representatives in 2017. A summary of the basic knowledge about various aspects of APP and the disease was subsequently published [2].

Since this review, considerable progress has been made, particularly in the areas of strain characteristics and disease pathogenesis. For example, only 16 serovars were known in 2018; today, 19 are known, and many atypical strains have also been described [3–5]. Although many virulence factors and their functions have been elucidated, virulence is still primarily explained by the Apx toxin profile. In a recent review, the capsular (CPS) and lipopolysaccharide (LPS) characteristics of APP biovars and serovars and their respective geographical distribution were summarised [6]. This knowledge is the basis for understanding serological cross-reactivity between different serovars and discrepancies between molecular and serological typing results. That review also discussed current knowledge on Apx toxins and some other virulence factors, as well as diagnostics and vaccines [6].

A recent review on APP vaccine development addressed how new-generation vaccines may overcome the limited cross-protection between APP serovars [7]. The potential of virulence factor-based subunit, live- attenuated, toxin-based, DNA and combined vaccines was discussed. A further review focused on the bacterial cell wall, adhesion, nutrient acquisition, and transport proteins, as well as on the isolation and identification of proteins in APP [8]. A high diversity of proteins is found in the outer membrane, outer membrane vesicles, and secreted proteins. Different functional proteins are decisive for mechanisms during different stages of infection and are therefore potential vaccine candidates.

These three APP reviews are an excellent source of knowledge on the bacterial composition, protein functions and host interaction at a molecular level. Less attention was given to a major knowledge gap, i.e., the prevention of disease under real field conditions; an area identified in the latest 2024 APP DISCONTOOLS survey [1]. Specifically, experts identified unresolved needs for control tools (diagnostics for colonized pigs, sufficiently protective vaccines, and therapeutics supporting eradication attempts). Other major knowledge gaps identified included a deeper understanding of immune responses, mucosal immunity, the impact of specific coinfections, persistence mechanisms and the impact of stressors. APP can rapidly adapt to stressors (e.g. exposure to antimicrobials), though its diverse defensome based on mobile genetic elements, including plasmids, prophages and integrative conjugative elements) [9].

As a primary pathogen, APP can cause severe disease without involvement of other factors. However, the starting point and transition process from a colonizing to an invasive pathogen are unknown. These are major research gaps crucial for effective farm management. The MISTEACHING (microbiome, immunity, sex, temperature, environment, age, chance, history, inoculum, nutrition, genetics) framework, which identified 10 factors decisive for disease pathogenesis in humans, was developed by Casadevall and Pirofski (2018) and, with the addition of strain (MISTEACHINGS), was shown to apply to APP [10, 11]. While only three factors strain, inoculum and microbiome were microbe-centric, five factors, genetics, age, immunity, nutrition, and history were host-centric. The three external factors, temperature, environment and chance, have additional impact on the course of disease, while the host-centric factor sex appeared to have no impact on APP disease [11]. Applying the framework of these 11 factors proved useful in identifying farm-related research gaps.

The recent DISCONTOOLS review, like the 2018 review [2], highlights an urgent need for new and improved strategies for prevention and therapy. Models for APP infection beyond in vitro models will be the basis for further innovation. The MISTEACHINGS framework led to the generation of new hypotheses, while the DISCONTOOLS framework aims to identify practical recommendations and associated knowledge gaps, enabling APP disease management on farms. This review article navigates between these two complementary approaches, focusing on the transfer of knowledge from the latest research findings on APP into agricultural and veterinary practice. This review, therefore, aims to complement recent vaccine- and serotyping-centred reviews by focusing on recommendations for diagnostics and practical disease control challenges in the field. Effective control will require a holistic approach through integration of herd management, antimicrobial stewardship, surveillance, and mechanistic understanding of bacterial and host factors. While vaccines and traditional diagnostics remain important, further focus should be given to the recognition of subclinical carriers and pathogen circulation on farms, impact of biofilm formation, regulation of virulence, immune evasion mechanisms, immunopathology, as well as molecular epidemiology. These topics are crucial for developing integrated approaches to control APP in contemporary pig production systems.

## Models for APP infection

Experimental APP infection models are essential for understanding the pathogenicity of APP and host interactions. While in vitro studies provide valuable mechanistic insights, they cannot replicate the complexity of porcine respiratory physiology, immune responses, or the multifactorial conditions of commercial production systems.

A major development since 2018 has been the refinement of experimental infection models with their different strengths and weaknesses for challenge studies assessing the virulence of poorly characterized serovars and highly virulent multidrug-resistant strains. These approaches provide a more realistic understanding of field strains compared with older laboratory isolates. For example, the significant pathogenic potential of a serovar 16 strain was demonstrated in an experimental piglet model [12].

In many studies, the natural host for APP, the pig, is used to identify new therapeutic measures and assess vaccine candidates [13–15]. Such a gold-standard approach determines if results apply to the field. Providing standard protocols are used, aerosol infection studies of pigs, for example, to assess vaccine candidates against many different serovars, are highly reproducible over time [16]. However, to carry out such experiments requires considerable expertise, time, and resources.

Although mice and greater wax moth (*Galleria (G.) mellonella*) are not natural hosts for APP, they are widely used as they are cheaper and more accessible than pigs (Table 1). A common approach is to undertake mouse experiments and, if successful, e.g., vaccine evaluation, validate the results in pigs - a cost-effective screening strategy. Mouse models, therefore, have a major role in assessing new therapeutics and vaccines and the understanding of APP-host pathogen- interactive biology.

**Table 1:** Research in models for APP infection 2018–2026

Research in mouse models
• Intraperitoneal and intranasal infection models in mice [17, 18]. • Infection of standardized, easily available, inbred BALB/c mice enabling comparability of trials in different laboratories [19, 20]. • Infection of easily available inbred C57BL/6 mice to study Th1 cellular immune mechanisms in response to APP vaccination and subsequent challenge experiments: APP-derived extracellular vesicles as a promising vaccine candidate due to the induction of APP-specific Th1-, CTL- and Th17-responses and stimulation of multifunctional T-cells [21]. • Infection of wild-type C57BL/6 and IL-21R-knock-out mice to determine the role of IL-21 in immune cell differentiation [22]. • Immunization of Kunming and outbred Institute of Cancer Research mice with APP-derived extracellular vesicles to detect differences in immune responses [18]. • Role of APP cAMP receptor in biofilm formation and colonization [23]. • Transcriptome analysis of biofilm-grown APP in a mouse model revealed repression of genes involved in carbohydrate and energy metabolism and up-regulation of genes contributing to exopolysaccharide synthesis and translocation [24]. • Aerosol mouse lung infection model to assess the therapeutic effect of ampicillin after APP infection: Inoculation of mice in an air-tight aerosol chamber with droplets (4–6 μm) generated over 5 min and inhaled for a further 20 min [25].
Research in greater wax moth (*Galleria* (*G*.) *mellonella*) models
• *G. mellonella* lacks an adaptive immune system, enabling specific host-pathogen interactions to be studied without the impact of acquired immune system responses [26]. • Characterization of the attenuation of APP *hfq* mutants in *G. mellonella* compared to the wild-type results in variation between different serovars [27]. Toxic effects of APP-derived extracellular vesicles in *G. mellonella* [28].

In addition, *G. mellonella* is a suitable, low-cost, rapid alternative infection model that can be used to compare the pathogenic potential of different APP strains and to evaluate the effectiveness of antimicrobial substances without the impact of an acquired immune system. Breeding conditions, nutrient availability, and environmental factors can affect the immune response of *G. mellonella*. Therefore, standardized rearing and testing protocols are necessary [26]. It is likely that, given the low cost, rapidity, and lack of ethical constraints associated with *G. mellonella*, there will be continued use of this model, particularly for novel APP antimicrobial screening and comparative virulence studies.

In summary, continued use of various APP infection models will lead to a greater understanding of APP and host interactions, allowing researchers to select a model appropriate for their needs. More head-to-head comparisons would be advantageous for the APP community in facilitating the choice of model to be used.

## Diagnostics

Recent reviews have described various aspects of APP diagnostics [6, 29]. The availability, sensitivities and specificities of diagnostic methods offered in diagnostic laboratories worldwide vary greatly. In general, the core methods used to diagnose APP infection, i.e., bacterial culture, real-time or molecular PCR (*apxIVA* and *apx*-toxin typing), and serology, are the same throughout the world. However, toxin typing appears to be more frequent in Europe and Asia, reflecting regional differences. For example, serotype 2 strains in North America typically produce ApxII, whilst those in Europe and Asia typically produce ApxII and ApxIII [30]. Thus, European and Asian laboratories include *apx* -toxin-typing in strain investigation to assess virulence potential. However, there have been recent reports of atypical toxin types in Korea [31], Japan, Chile, the USA [32] and Europe [33], and this is likely to drive further worldwide use of *apx*-toxin typing as a diagnostic and epidemiological tool. The recent period (2018–2026) has seen incremental improvements as well as some innovations, but also persistent challenges in diagnostics (Additional file 1).

The availability of centralized (e.g. EU) registration procedures and a database for diagnostic kits would be a valuable resource for practitioners, so that efforts in this area should be strengthened. Non-typable or atypical APP strains should be characterized and collected in a reference laboratory. An observed correlation between biotype, serotype and virulence in one geographical region cannot necessarily be extrapolated to another region.

In most cases, clinical disease is caused by a single serovar. However, especially after mixing pigs from different farms, more than one strain can be involved in the outbreak or isolated from healthy carrier animals [34]. Diagnostics should therefore include various animals and further typing of different APP colonies. APP isolates of the same serotype but with different antimicrobial resistance patterns were obtained from a farm on the same day [35].

Bacteriological diagnosis using fresh lung samples from untreated animals is mostly successful [6, 29, 35]. Bacteriological examination of tonsillar tissue is hampered by commensal bacterial flora frequently outgrowing APP [6, 29]. Low-virulence APP strains may colonize the tonsils without causing clinical disease [6]. Experimental infection studies have shown that in cases of “multi-organ spreading” with case reports of arthritis, osteomyelitis, hepatitis, meningitis or nephritis, alternative samples may also be useful for detecting APP [36]. Pleuritis or dorsal pleuropneumonia recorded during slaughterhouse lung checks scores are surrogate markers for APP herd problems. The percentage of altered lungs is a good basis for decisions on therapy and prophylaxis [37], but aetiological diagnosis should be performed in parallel.

In chronic or subclinically infected pigs, tonsil examinations using culture and PCR have proven effective, although false-negative results cannot be ruled out. In addition, antibodies against ApxI-ApxIV, LPS, CPS or surface proteins should be measured, although cross-reactions or unexplainable results can occur [6, 38]. Selected commercial APP vaccines available in Europe and some Asian countries led to positive results in several commercial ELISAs, but also to a functional immune response measurable by opsonophagocytic antibodies [39].

Biochemistry and serology are no longer considered adequate for identifying APP at the species level due to atypical variants [6, 40, 41]. Aberrant APP biovar 1 (NAD-dependent) and 2 (NAD-independent) strains with respect to serovar have been reviewed by To et al. [6].

Accurate typing of APP isolates is crucial for understanding the epidemiology of outbreaks, monitoring infected herds, and preparing or selecting vaccines for disease control, as the efficacy of widely used bacterin vaccines is mainly serovar-specific. Frequent divergent CPS and LPS combinations led to cross-reactions between serotypes such as 1, 9, and 11, and 3, 6, 8, 15, and 17, complicating differentiation by agglutination with specific sera [6]. The *apx* toxin gene pattern of atypical isolates is not concordant with its derived serotype, capsule genes of a strain are controversial to its LPS biosynthesis genes [5, 32]. Although mPCR-based precise serovar identification techniques enabling differentiation of all 19 known serovars were developed, the emergence of atypical strains will require additional or at least combined typing strategies in the future [4, 42–45]. The availability of multiple whole-genome sequences, molecular biological methods, and MALDI-TOF-MS combined with appropriate databases, will allow the bacterial species to be sufficiently confirmed [5]. Using specifically prepared filter paper (Flinders Technology Associates (FTA) technology) FTA^®^ cards, infectious agents (viruses, bacteria, fungi, parasites) can be inactivated, and their DNA and RNA stabilized. The proprietary reagent contains a weak base, a chelating agent, an anionic surfactant, and a free radical scavenger [46]. After reconstituting the samples, multiplex PCRs (mPCRs) can simultaneously detect the presence of APP, the serovar(s), and biovars present, even in samples derived from pig lungs [29, 47].

Third-generation sequencing technologies combined with bioinformatic tools can facilitate typing and antimicrobial characterization of APP [42, 48–50].

If sequencing options become even more accessible and cost-effective, whole-genome sequencing will likely become the method of choice for the comprehensive typing and characterization (serotype, virulence markers, antimicrobial resistance) of APP isolates in the future. Diagnostic approaches should include covering under-characterized or newly regionally emerging strains, which may harbor novel virulence or resistance profiles [12].

In the future, real-time nucleic acid sequencing platforms (e.g. Nanopore) have major potential for use in point-of-care diagnostics, as coinfecting agents, which can be major trigger factors for disease outbreaks, can be simultaneously detected [51–54].

## Prevention of disease

Not only results from field studies, but also from basic research have implications for disease prevention (Table 2). A key difficulty in controlling APP is the discrepancy between acute outbreak management and long-term herd persistence. In many cases, clinical outbreaks may be successfully contained through antimicrobial intervention and supportive management, yet the pathogen remains present in the population. Subclinical carriers serve as reservoirs, allowing re-emergence of disease months or even years later.

**Table 2:** Research results with implications for disease prevention 2018–2026

Breeding for naturally occurring genetically defined disease resistance
• Quantitative trait loci (QTL) on 13 chromosomes for APP serovar-specific humoral immunity [55]. • QTL on SSC14 associated with surface IgM complexes against APP serovars 3, 5, 7 and 13 can be hypothetically used in commercial breeding programs to improve resilience [11]. • Genome-wide association studies in pigs susceptible and resistant to APP after experimental challenge identified SNPs on SSC2, SSC12 and SSC15 explaining > 50% variance in disease symptoms [56].
Reduce mixing of pigs and animal movements within and between farms
• APP serovar 15 was detected up to six months post-outbreak in subclinically infected pigs, contributing to ongoing transmission [57]. • Whole-genome sequencing of APP outbreak strains identified high-risk clones with antimicrobial resistance genes and classical pathogenicity determinants, enabling rapid spread in herds under antimicrobial pressure [58]. • Genomic analyses of persistent strains revealed horizontal gene transfer, suggesting that specific clones are highly adapted to the porcine respiratory tract [58–60]. • Vertical transmission of resistant APP phenotypes in pig populations needs identification of epidemiological links (e.g. between age groups) to optimize antimicrobial usage [61].
Control of coinfecting agents
• APP coinfection with swine influenza A virus has a high impact, and with PRRSV has less impact on disease severity [62, 63]. • APP infection and disease are frequently complicated by *M. hyopneumoniae* and *Pasteurella multocida* [63–67]. • Given that APP is a primary pathogen of the PRDC, it is unknown whether specific genes of APP are required for survival when the infection is mixed with viral infection (or even other bacteria). For example, it was shown that specific genes of *Haemophilus influenzae* are required for survival in mice co-infected with influenza A virus but not for bacterial infection alone [68]. • APP strains of lower virulence can cause severe disease in case of immunosuppressive viral infections (e.g. porcine circovirus-2, PRRSV). [67, 69, 70]. • SIV circulating subclinically in young pigs can potentially trigger APP infection [71]. • SIV can primarily cause severe disease with later complications by APP [71]. • APP might benefit from coinfection with *S. suis* in the lung, but the mechanisms involved are unknown [72–74]. • Colocalization of APP and *S. suis* in biofilms might have a significant implication for persistence and pathogenicity of both pathogens [73]. • APP and *S. suis* form two-species biofilms in vitro without NAD supplementation [74]. In the co-culture model, antimicrobial resistance and upregulation of virulence-associated genes in both APP and *S. suis* were increased.
Avoid suboptimal stable climate
• Frequent APP outbreaks in winter followed by autumn [75] reflecting impact of season, ambient temperature and humidity on disease outbreaks in farms endemically infected with APP [75, 76]. • NH_3_ induced APP biofilm dispersal and planktonic growth with a modulation in gene expression [77]. Calculation example: 20 ppm (≈14 µg/L ≈ 0.8 µmol/L) legal threshold for air NH_3_ in swine housing in Germany. Impacted dust-bound NH_3_ (3 µg/mg stable dust [78] leads potentially to increased local NH_3_ in the epithelial lining fluid of the respiratory tract (600 mL tidal volume in a pig (60 kg body weight) [79] ⇒ 12 L respiratory volume per minute, 5 mg/m^3^ indoor air dust concentration [80] ⇒ 250 µg NH_3_ soluted in 1 ml epithelial lining fluid within 24 h ⇒ 15 mmol NH_3_ /L, which is above 10 mmol NH_3_/L tested in vitro [77].
Perform susceptibility-guided antibiotic treatment
• Differences between countries in antimicrobial resistance rates against various antimicrobial substances [81]. • Increasing resistance rates against aminoglycosides (streptomycin and gentamicin) in Romania [82] • Increasing resistance rates against aminoglycosides, tetracyclines, macrolides and quinolones [49, 58]. • Emergence of quinolone-resistant APP-strains, including a decrease in drug influx and an increased drug-pumping ability (drug efflux). Efflux pumps actively expel antibiotics from bacteria, while mutations in target sites, such as *gyrA* and *parC*, can also confer quinolone resistance and a weakening of drug binding by a mutation in the resistance-determining region [83]. • Detection of florfenicol resistance in APP serovar 6 in Italy and several Chinese strains due to the presence of the *floR* gene located on plasmids suggesting horizontal transfer. Plasmid pAp-floR has high nucleotide identity and coverage with the pMVSCS1 plasmid (5621 bp) from *Mannheimia varigena* MVSCS1 with probably no fitness cost to APP (stable irrespective of selection pressure) [84, 85]. • Multidrug-resistant isolates associated with specific serotypes in Italy [86]. Frequent serotype 9/11 was associated with resistance to enrofloxacin and florfenicol [86]. • Multidrug resistant APP serovar 1 with atypical toxin gene pattern causes high losses [87].

Strict protocols for animal introduction, pen segregation, and environmental sanitation reduce the spread of APP, particularly by persistent carriers. Age- or group-specific housing minimizes exposure of susceptible pigs to chronic carriers. The convergence of antimicrobial resistance, biofilm-mediated persistence, and immune-mediated pathology has several direct implications for swine producers:

### Long-term surveillance and emerging serovar monitoring

Diagnostic screening, regional surveillance networks, and careful monitoring of subclinical carriers to limit chronic infection reservoirs are of high importance for the prevention of disease outbreaks [57].

For confirmation of an APP-negative status in a herd, as well as for herd surveillance and assessment of the infection cycle in endemically infected farms, more than one diagnostic method should be combined, particularly for detecting low-level carriers. So far, no general guidelines for diagnostic procedures to test the absence of APP infection to enable certification of herds as specific pathogen-free (SPF) with respect to APP exist. Sample size estimation should depend on statistics and study purpose [88]. To identify a farm as positive, the sample size might be approximately 30 individuals in the age-group with the highest probability of being positive (assuming a 10% prevalence and 95% statistical confidence). A general procedure in case of (false) positive test results in a farm with SPF status for APP is needed. Assessment of the colonization status of pigs in different age groups might require a large sample size [88]. The statistically necessary sample sizes can be reduced by a combination of methods, such as tonsillar scrapings analyzed by *apxIVA*-based PCR with serological testing using ELISA [29]. In the future, expensive PCR testing of tonsillar scrapings may be replaced by cheaper methods, such as LAMP or other rapid amplification-based point-of-care tests [89, 90]. In comparison to tonsillar samples, positive nasal swabs might indicate a period of pathogen transmission.

### Integrated herd management

Practical control measures for disease prevention, such as strict biosecurity, quarantine of new animals, age group and barn segmentation, are of high importance to reduce transmission routes [57]. APP biofilm formation during colonization is considered a strategy for immune evasion and persistence in the host [77]. Recently, genomic analyses of persistent APP strains in carriers revealed horizontal gene transfer, suggesting that specific clones are highly adapted to the porcine respiratory tract [58–60]. APP anaerobic genes identified in a Selective Capture of Transcribed Sequences (SCOTS) approach were found to be decisive for long-term colonization (40 days) of pig lungs [91]. While planktonic bacteria may drive acute disease, biofilm-associated populations may support chronic colonization and later reemergence of the pathogen [24]. Also, the host genetic background has a high impact on resistance or susceptibility to APP, and the identification of important genetic traits has the potential for use in herd health stabilization approaches [11, 55, 56].

From a practical standpoint, this persistence complicates decisions regarding depopulation, strategic medication, and vaccination timing. Herds may experience repeated production losses due to chronic pleuritis detected at slaughter, even in the absence of severe clinical pneumonia. Thus, APP represents not only an acute health emergency but also a chronic productivity constraint.

Longitudinal studies indicate that APP can circulate within herds for months after clinical outbreaks. Colonization of piglets starts during the suckling period, but there is a lack of knowledge about the transmission of APP generally and specifically from sows to their offspring in the farrowing room. To date, no interventions are known that effectively reduce sow-offspring transmission and colonization in suckling piglets. Sow vaccination resulting in high maternal antibodies and a reduction in colonization of suckling piglets is an obvious approach. However, no such effective vaccine has been reported.

High direct transmission rates occur after weaning in the nursery unit and can be followed by disease outbreaks, frequently in weeks 10–12 of life. Epidemiological factors influencing disease spread within the herd include herd size and various aspects of internal biosecurity, such as high stocking density, inadequate ventilation, stress from mixing age groups, vaccination status, and the presence of other respiratory pathogens predisposing pigs to clinical pleuropneumonia [2, 11].

Measures that reduce the infection pressure of coinfecting agents of the porcine respiratory disease complex (PRDC) can sometimes be effective in preventing recurrent APP outbreaks. Coinfections and other trigger factors are hypothezised to be responsible for disease outbreaks also in vaccinated herds. Knowledge of specific APP genes required for polyinfections (e.g., APP-influenza, APP-PRRSV) is necessary to unravel the pathobiology of coinfections in the future.

Recent studies underscore the need for integrated approaches that combine improved housing conditions with pathogen monitoring. Empirical observations support frequent outbreaks under cold weather conditions, when transmission is facilitated by the huddling behaviour of pigs [75, 76]. Environmental ammonia is considered a further risk factor for disease outbreaks [77]. Whether APP persistence in tonsils is enhanced by growth in biofilms, and external environmental signals as NH_3_ induce dispersion with subsequent invasive infection, is unknown, but worthy of study.

### Vaccination

APP can be classified as a damage response framework (DRF) class 2 pathogen [11], since it induces tissue damage in hosts with weak or normal immune responses [92]. In theory, vaccination is therefore a promising prevention strategy. Current commercial vaccines are effective in reducing clinical signs and lung tissue alterations, but do not prevent colonization, biofilm-associated persistence and transmission. Accompanying measures targeting biotic and abiotic trigger factors might improve the efficacy of vaccination protocols based on commercial whole-cell-, subunit- or autologous whole cell vaccines.

In general, there is a need for vaccines that do not interfere with or are impaired by maternal antibodies present at a very young age and are efficacious up to slaughter. Therefore, the optimal pig age for APP vaccination is still under discussion and varies between different herds. One goal would be the creation of a vaccine that can be used in the suckling period without interfering with maternal immunity. Measurement of maternal antibody levels against Apx toxins, which potentially interfere with piglet vaccination, would be valuable.

Many different vaccine approaches, e.g., recombinant, live attenuated, outer membrane vesicles, mRNA, whole-cell genetically modified, etc., are being pursued to address the limitations of current vaccines. The topic will not be considered in detail here as a comprehensive review of APP vaccines and development strategies has recently been published [7].

## Treatment

### Antibiotic therapy

Therapeutic intervention of diseased pigs with antimicrobials is mostly successful, but does not prevent long-term persistence of APP in the tonsils. The metabolic and morphological changes observed in biofilm-associated APP suggest that this phenotype may influence pathogenic potential in vivo as well as treatment success [24, 93]. Generally, biofilm formation is considered a strategy for immune evasion (protection from phagocytosis and antibodies) and persistence in the host, hampering effective therapy [24, 77]. Novel therapeutic approaches may be required to inhibit APP biofilms, and such studies identify potential targets for novel antimicrobials (Table 3). For more effective therapy, the dispersal of biofilms, e.g. on the tonsils or in lung tissue, should be addressed in future therapeutic approaches [29]. In addition to growth in biofilms, the intracellular survival of APP in macrophages can limit treatment effects. Bacterial adhesion by the Adh protein belonging to a trimeric autotransporter family led to an increased LPS secretion inducing the overexpression of host cation transport regulatory-like protein 2 (CHAC2). As a consequence, a reduced phagocytosis capacity and increased intracellular survival in pulmonary alveolar macrophages via the LPS-TLR4-CHAC2 pathway led to a reduced treatment efficacy [94].

**Table 3:** Research results with implications for treatment and eradication 2018–2026

Antibiotic treatment
• High efficacy of ceftiofur (epidemiological cut-off for APP: 0.125 µg/mL, minimal inhibitory concentration (MIC) 0.5 (in vitro) and 1 µg/mL (ex vivo) [95]. This was in accordance with CLSI cut off of ≤ 2 µg/mL and MIC90 of 1 µg/mL [96]. The recommended optimal bactericidal ceftiofur concentration for APP was 3.21 mg/kg to achieve MIC90 [95]. • Recommendation of 20–30 mg benzylpenicillin/kg body weight in an oil-based suspension administered every 12 h [14]. • Adapted pharmacodynamic ratios for trimethoprim/sulfonamide to optimize synergy in bacterial killing rates [97].
Potential alternative therapeutic substances
• H_2_O_2_ (0.5 mM) produced by the common nasal colonizer *Streptococcus plurianimalium* during aerobic metabolism is toxic for planktonic APP cells in vitro [98]. The bacterial metabolite H_2_O_2_ can induce biofilm formation of specific bacteria, but can also increase bacterial sensitivity to antimicrobials by shifting the balance between pro- and antioxidants in the bacterial environment [99]. • Rhein (4,5-dihydroxyanthrachinon-2- carbonic acid) obtained from rhubarb species (*Rheum palmatum*, *Rheum officinale*) has anti-inflammatory properties, killed APP (MIC 25 µg/mL) and reduced biofilm formation in a mouse model [100]. • Herbal-derived feed additives quercetin and epicatechin gallate prevented damage to porcine alveolar macrophages and increased expression of important inflammatory cytokines after APP infection [101]. • Flavonoid naringin from the pericarp of rutaceae fruit relieved severe lung injury caused by APP via inhibition of the MAPK/NF-κB signaling pathway, exerting an anti-inflammatory effect [102, 103]. • Essential oil eugenol was effective against APP in vitro (0.49 mg/mL) [104]. • The anti-diabetic substance halicin exhibited killing and long post-antibiotic effects against APP without resistance development within 40 days at sub-inhibitory concentrations [105]. • Tea polyphenols inhibited the growth of APP, decreased adherence to tracheal epithelial cells, attenuated the release of pro-inflammatory cytokines and improved the survival rate of APP-infected mice [106]. • Efflux pump inhibitor Carbonyl Cyanide Chlorophenylhydrazone restored the antibacterial effects of florfenicol in resistant APP strains [107].
Eradication protocols
• Medicated early weaning (day 9 of age) with sow and piglet medication was not successful in raising only negative pigs [108]. • Removal of pigs < 10 months of age and intramuscular treatment two to three times in three-day intervals of all remaining pigs with enrofloxacin (7.5 mg/kg body weight), accompanied by cleaning and disinfection of empty units [109]. • Prevention of piglet colonization with APP and *P. multocida* by intramuscular sow treatment with the macrolide tildipirosin once after farrowing and suckling piglets three times in 7-day intervals starting on the day of birth [110].

Various classes of antibiotic substances are successfully used to treat infection with APP, including also critically important antimicrobials (Table 3) [29]. Some authors consider benzylpenicillin to be the drug of choice for treating APP infection [14]. Clinical breakpoints approved by the Clinical and Laboratory Standards Institute (CLSI) for most antimicrobial substances are unavailable or require re-evaluation [96]. For example, there are no breakpoints for penicillin and cephalothin, and those for fluoroquinolones need re-evaluation [111].

Most practitioners consider antibiotic resistance as a concern rather than a major practical issue, as at least in Europe, APP is susceptible to antimicrobials in general use when tested under in vitro conditions [81, 86, 96, 112, 113]. However, the occurrence of high-risk APP strains carrying genes for antimicrobial resistance and virulence highlights the limitations of empirical therapy and the urgent need for integrated genomic epidemiology, allowing early detection of dangerous clones before they become widely disseminated, as well as susceptibility-guided antimicrobial administration [58, 59]. Such strains represent a dual threat: they are both difficult to treat and capable of causing severe clinical disease [58, 114].

APP antimicrobial resistance can be mediated by a multifactorial rather than a single genetic determinant. For example, efflux-mediated resistance may confer cross-resistance across multiple antimicrobial classes, accelerating the emergence of multidrug-resistant phenotypes.

Antimicrobial resistance patterns vary from country to country and likely reflect different patterns of antimicrobial use. Increased usage appears to be associated with increased resistance. For example, high-level resistance to historically widely used tetracyclines and doxycycline has been observed worldwide [31, 35, 50, 86, 115–117]. Resistance to florfenicol is widespread in Korea [31], China [116] and Taiwan [35], but is rarely reported in Europe or the Americas. Notably, an increase in florfenicol resistance in Italy was accompanied by a 26% increase in amphenicols sales, although it was not possible to directly link the two observations [86]. A direct linkage between usage and resistance is not always possible as overall usage and treatment patterns, e.g., dosage and treatment periods, may differ [118].

No association studies between antibiotic usage and antimicrobial resistance rates in APP exist, although antimicrobial sales data and antimicrobial resistance rates are available from different countries [81, 113, 119]. In a recent European survey covering the time period 2019–2020, antibiotic resistance in respiratory pathogens was similar in magnitude to previous time periods but with still high variations in resistance rates, especially for tetracyclines and quinolones [81]. Studies about antimicrobial resistance rates in APP for 2018–2026 are summarized in the Additional file 2.

In the future, herd-specific susceptibility testing, rotation of antimicrobial classes, and restriction of critical drugs are likely to become routine to reduce the selective pressure of antimicrobial substances [58, 82, 83, 86].

### Future alternative treatment strategies

The emergence of APP strains with antimicrobial resistance, as well as the presence of multiple APP serotypes, with a risk of reduced vaccine cross-protection, has led to increased interest in the use of natural products that alleviate oxidative stress and inflammatory signalling pathways during APP infection. Anti-inflammatory or oxidative stress-targeted therapies may reduce APP-induced acute lung damage during acute outbreaks, potentially lowering mortality and improving recovery [102, 103, 120, 121]. Other, alternative therapeutic approaches, e.g., immunomodulatory, probiotic and plant-based therapies are not yet standard in veterinary practice, but are the subject of ongoing research due to their potential role in APP control (Table 3). One approach to overcome antibacterial resistance is to use a combination of antimicrobial substances with efflux pump inhibitors. This adjunct therapy can restore susceptibility towards antimicrobial substances also in APP [107].

A further focus is on the more effective killing of APP in biofilms. Dispersin B and polymeric β-1,6-linked N-acetylglucosamine (PNAG) are involved in biofilm formation [24, 122]. For more effective therapy, the dispersal of biofilms, e.g. on the tonsils or in lung tissue, should be addressed in future therapeutic approaches, addressing also the risk of the higher virulence of planktonic cells [29]. An elegant engineering approach to disrupt PNAG-dependent biofilms was to combine phage delivery of dispersin B via phage, enabling high concentrations of enzyme and lytic phage [123]. Even small initial inocula of the engineered T4 bacteriophage disrupted the *Escherichia coli* biofilm. However, although bioinformatic analyses have indicated the presence of prophage in APP [9, 60], the only experimentally confirmed active phage described is a Mu-like bacteriophage called PluMu [124]. Being a temperate, rather than lytic phage, and of restricted infectivity (serovar 1 and 11 strains), PluMu is not suited for bacteriophage therapy. However, future genome and experimental approaches may identify a broad APP lytic phage that could be engineered for biofilm dispersal.

An in silico subtractive/comparative genomics approach was used to identify 122 essential proteins, 95 of which were cytoplasmic and 11 transmembrane, suitable as drug and vaccine targets, respectively [125]. However, further experimental validation is required to determine whether such targets can be translated to the clinic.

In addition, our understanding of the host response to APP infection has identified pathways whose targeting may be suitable for treating sick animals, including IFN-γ [126], and silent information regulator 2 (SIRT2) [127]. We envisage that future transcriptome, proteome, and metabolic modelling-based studies will identify more targets for therapeutic interventions for APP disease. Such studies are complementary to those aimed at determining the mode of action of natural products.

Future therapies may also be based on antimicrobial peptides, e.g. Mastoparan X (MPX), a 14-amino acid peptide belonging to the wasp venom AMP family, that was synergistic with tulathromycin against APP [128]. MPX had previously been shown to significantly reduce biofilm formation in vitro and protect mice against a lethal dose of APP [129]. PR-39 is a linear 39-amino acid natural antimicrobial peptide in pigs. Transgenic mice expressing PR-39 were partially resistant to APP infection [130]. However, expression of APP SapA confers resistance to PR-39 [131] and MPX [129]. Overall, the results show that antimicrobial peptides have potential as new therapeutic agents, particularly in combination with existing antibiotics.

Advances in artificial intelligence (AI) also led to the discovery of new potential antimicrobial drugs from a collection of more than one billion chemical substances [105]. In a more targeted approach, various short- and medium-chain organic acids and essential oils were tested with varying efficacy against APP in vitro [104].

## Eradication

Currently, there is no foolproof strategy to eliminate APP from the tonsils, which hampers sanitation and eradication programs. Efforts in endemically infected herds are made to stabilize animal health to prevent disease outbreaks. This includes the avoidance of stressors such as mixing, transport, or coinfection with viral pathogens.

Stock replacement by APP-free animals is considered the gold standard for eradication. To verify that a herd is free of APP is impossible, but the probability can be increased by tonsillar (scraping or brushing) sampling a high number of animals for PCR diagnostics in combination with serological tests (see Long-term surveillance and emerging serovar monitoring).

APP eradication protocols and experiences are rarely published, possibly reflecting the lack of success. Published eradication protocols are based on a combination of removal of susceptible pig groups, vaccination and antimicrobial treatments with substances reaching high concentrations in tonsillar tissue (Table 3). The intense antimicrobial treatment has the potential to select for resistant bacteria [109].

## Conclusion

APP should be considered as an adaptable pathogen capable of persistence, immune evasion, and endemic circulation in modern pig populations.

Future control strategies will require integrated approaches that combine antimicrobial stewardship, surveillance of virulent multi-drug- resistant clones, biofilm-targeted interventions, and a deeper understanding of mucosal immunity and carrier states. By integrating these strategies, veterinarians and producers can reduce morbidity, mortality and economic losses associated with APP.

Future research priorities should address the following gaps:

1. Mechanistic biofilm studies
  Detailed mechanistic studies on gene regulation, matrix composition, and metabolic adaptation in APP biofilms could lead to the development of novel therapeutic strategies.
2. Host immune interactions
  Detailed investigation of mucosal immunity, cytokine signaling, and immune evasion mechanisms is required to develop interventions that prevent chronic colonization and immunopathology. Next-generation vaccines targeting conserved surface antigens and biofilm-associated proteins may offer improved protection. Understanding local immune responses in the respiratory tract will aid vaccine development that elicits effective mucosal protection.
3. Genomic surveillance integration
  Broader implementation of whole-genome sequencing in field strains can identify high-risk clones with both virulence and multidrug resistance traits, improving outbreak prediction and control.
4. Alternative therapeutic strategies
  Adjunct treatments targeting biofilms, immune modulation, or oxidative stress should be tested under field conditions, potentially reducing reliance on conventional antimicrobials. Development of anti-biofilm agents, immunomodulators, and phage therapy may complement traditional antibiotics.
5. Cross-disciplinary herd management
  Veterinary herd health management will include information from outbreak epidemiology, genomics, immunology, and pharmacology for the formulation of integrated approaches for long-term APP control, including strategic vaccination, biosecurity, and treatment protocols.

## Supplementary Information

Additional file 1: Diagnostic approaches for APP infection. Content: Literature research results to summarize the gain in knowledge 2018-2026 for the diagnostics of APP infection compared to basic diagnostic procedures.

Additional file 2: Antimicrobial resistance in APP 2018-2026. Content: Literature research results on published antimicrobial resistance rates in different countries. Intermediate isolates were grouped with the resistant ones if information was available. CLSI cut-offs were applied wherever possible to improve comparability between studies. Data shown in this Table were calculated from the published datasets, if necessary. *Data were retrieved from recorded references and adapted by the authors to improve comparability between published studies [132–161].

## Data Availability

This review is based on the Discontools database on research gaps for improving infectious disease control in animals (https://www.discontools.eu/database.html, last accessed: 3rd June, 2026).
