# Supplementary material for: Actinobacillus pleuropneumoniae – today's perspective on advances and challenges (2017- 2026)
Source: BMC Vet Res. 2026 Aug 11;22:466. doi: 10.1186/s12917-026-05791-3 (PMC13465399; doi:10.1186/s12917-026-05791-3)
Supplement: Supplementary file 1 [file 12917_2026_5791_MOESM1_ESM.docx]

**Additional file 1.** Diagnostic approaches for APP infection

| **Basic diagnostic procedure** | **Gain in knowledge 2018-2026** | |
| --- | --- | --- |
| **APP isolation and culture**  Advantage: APP isolates can be used for further characterization (serotyping, *apx*-toxin typing, antimicrobial resistance testing, whole genome sequencing, etc.) | | |
| - Gram staining of smears from lung lesions to detect Gram-negative rods. - Bacteriological examination of affected lung tissue on 5% sheep blood agar with *Staphylococcus epidermidis* or *Staphylococcus aureus* nurse for V factor (NAD), resulting in a hemolysis zone (37°C with 5% CO_2_). - Chocolate agar or PPLO agar supplemented with NAD, yeast extract or other supplements (e.g. PolyViteX by bioMérieux; IsoVitaleX by BD BBL) with APP colonies being less distinctive [6, 29, 35]. - Two APP biovars, based on NAD requirement for growth: biovar 1 (NAD‐dependent: 17 serovars (1−12 and 15−19)), biovar 2 (NAD‐independent: 8 serovars (2, 4, 7, 9, 11, 13, 14, and 17)) [6]. - APP isolation from tonsils and nasal cavity by enrichment with immunomagnetic separation [132]. | - Culture: overall sensitivity of 88.0% in lung tissue seven days after experimental infection [133]. - NAD‐independent (biovar 2) isolates harbor full‐length functional *nadV* gene (1479 bp) encoding nicotinamide phosphoribosyl-transferase for utilization of NAD precursor nicotinamide in their environment [36, 133]. NAD‐dependent (biovar I) isolates harbor an inactive truncated *nadV* gene (612, 615, or 618 bp) [5, 36, 41]. - NAD dependence can be determined by a specific PCR (target gene was integrated into APP-mPCR, frequently used for CPS-typing of APP [3, 4]. - Incorrect typing of APP biovar 2 strains as *A. suis*, which cause similar lung alterations [6, 134]. - Diagnostic sensitivity (84.7%) and specificity (66.7%) of tonsillar APP isolation seven days after experimental infection (predictive values: positive 94.6%, negative: 35.3%) [133]. | |
| **Molecular methods** | | |
| - PCR with species-specific target genes (*apxIVA*) in various sample types [6, 29]. | - Real-time PCR (*apxIVA*) in oral fluids suitable for APP non-invasive surveillance [135]. - Tonsillar samples from carrier pigs are superior to nose samples for quantitative PCR [55]. - Molecular typing and routine qPCR testing of emergent APP strains before onset of disease [48, 55]. - APP species confirmation from colony material by MALDI-TOF-MS (depending on database) [5]. - APP PCR from samples collected on FTA^®^ card (long-term storage, protection from RNA-/DNA degradation, no risk of biohazards) [47]. - Multiplex-PCR to differentiate APP from *Glaesserella australis* and *Pasteurella multocida* [51]. - TaqMan duplex real-time PCR for simultaneous detection of *Streptococcus suis* and APP in mixed biofilms [52]. - Triplex real-time PCR for *S. suis*, *Glaesserella parasuis* and APP [53]. - Point-of-care testing (duplex TaqMan real-time PCR) for APP and influenza A virus based on probes and primers for *apxIVA* gene and conserved matrix gene of H1N1 [54]. - Isothermal amplification techniques (*apxIVA*) for rapid testing in clinical settings [57, 58, 136-138]. - *apxIVA*-based loop-mediated isothermal amplification (LAMP) assay compatible with Dragonfly platform [139] (www.protondx.com) for colorimetric detection of APP in lung samples within 30 minutes [140]. | |
| **Typing of APP (serotyping)** | | |
| - Serological typing using specific anti-sera for agglutination based on CPS, typically associated with a particular LPS type. - Failure of serovar-specific agglutin-ating sera in serotyping of non-encapsulated APP (insertion of IS*Apl1* into CPS biosynthetic gene led to defect in capsule production [141]. - *apx-*toxin gene-based PCR for assessment of virulence of a strain [5, 33]. - Proposal of hybrid serovar 9/11 due to the same O-antigenic locus and toxigenic profiles (one base difference in the *cpsF* gene). | - mPCRs based on CPS-/O-antigen encoding target genes specific to each serovar, as well as the species-specific *apxIVA* gene [4, 43-45]. - 12 highly conserved DNA marker candidates for identifying APP with potential mutations in existing markers (e.g. *apxIVA* gene) [142]. - Recommendation for *apx-*toxin gene-based PCR because of the occurrence of isolates with atypical *apx* toxin gene profiles has increased [5, 33]. - qPCR for differentiation between European serovar 9 and 11 [143]. - Atypical serovar 12 strains of high virulence (ApxII and III production, LPS biosynthesis genes of serovar 15 [32]. - Atypical serovar 1 strain with defect in capsule production [144]. - Detection of atypical serovar 12 (as determined by PCR) strains that do not express capsule [145]. - Bioinformatic tools assigning the serovar from whole genome sequences (https://github.com/ KasperThystrup/ serovar_detector) [42, 48]. - Whole genome sequencing as a predictor for antimicrobial resistance (except for the macrolides) [50]. - Good agreement between phenotypic and genotypic sensitivity to florfenicol, tilmicosin, doxycycline, and oxytetracycline [49] | |
|  |  |  |
| **Serological assays** | | |
| - Highly specific ApxIVA‐based ELISAs for serological screening (DIVA strategy) [146]. - APP field isolates with insertion sequences within the *apxIVA* gene (e.g., IS*Apl1*) generate false negative serological results [147]. - Asymptomatic colonized pigs stay negative for ApxI- and ApxII-specific antibodies [148]. - Differentiation of serovar groups (1‐9‐11, 2, 3‐6‐8‐15‐17, 4‐7, 5, 10, and 12) by commercial LPS-ELISAs (e.g. Biovet, Canada; IDVet, France, Biochek, The Netherlands), but cross-reactions can occur [149]. - Combination of LPS‐based ELISAs had higher sensitivity than an ApxI- and an ApxIV-based ELISA [150]. | | - Recommendation for lower cut-off of 10 in commercial ApxIV-ELISA (IDEXX APP-ApxIV Ab Test, IDEXX Laboratories, Westbrook, ME,USA) to avoid false-negative results [151]. - Commercial ELISA (App OmpA ELISA, BioChek, The Netherlands) in combination with ApxIV-ELISA for assessment of active and maternal antibodies. Low level of maternally derived OmpA antibodies in the presence of higher levels of ApxIV antibodies hypothetically indicates a low risk of interference with maternal immunity after piglet vaccination. [152]. ([https://www.biochek.com/ swine/app-elisa-test-system/](https://www.biochek.com/%20swine/app-elisa-test-system/), accessed 22.7.2026). - Multi-parameter IgG screening test to detect APP (LPS-antigen) among other pathogens in meat juice or serum with moderate accuracy (0.77), low sensitivity and specificity at a chosen cut-off (0.59) in a ROC-analysis [153]. |
| - Microsphere-based multiplex fluorescent immunoassay (MFIA) based on fluorescently barcoded microspheres bound to serotype‑specific antigens (Luminex Multi-Analyte Profiling of analyte x (xMAP) technology) allows the simultaneous differentiation of antibodies against different APP serotypes in a single well [154]. | - ApxIV-based ELISA with lower sensitivity (89,4%) than LPS-ELISA and (MFIA), so that the ApxIV ELISA should be combined with other methods when a negative APP status of a population must be confirmed [151]. | |
