# Supplementary material for: Actinobacillus pleuropneumoniae – today's perspective on advances and challenges (2017- 2026)
Source: BMC Vet Res. 2026 Aug 11;22:466. doi: 10.1186/s12917-026-05791-3 (PMC13465399; doi:10.1186/s12917-026-05791-3)
Supplement: Supplementary file 2 [file 12917_2026_5791_MOESM2_ESM.docx]

**Additional file 2** Antimicrobial resistance in APP 2018-2026

|  | Amoxicillin (Am) Ampicillin (A) | Ceftiofur | Enrofloxacin | Forfenicol | Macrolides:  Tulathromycin (Tu)  Tilmicosin (TM)  Tylosin (T) | Penicillin | Tetracyclines (T) Oxytetracycline (O) Doxycycline (D) | Tamulin | Trimethoprim-Sulfamethoxaczol | References^*^ |
| --- | --- | --- | --- | --- | --- | --- | --- | --- | --- | --- |
| China (2019-2021), Disk diffusion method  N=28 | 32.1 (Am) 53.6 (A) | 7.1 | 60.7 | 64.3 | 96.4 (TM) | 64.3 | 89.3 (T) 17.9 (D) |  | 85,7 | [155] |
| Poland (2019-2024), Disk diffusion method  N=119 | 0 (A) | 7.6 | 0 | 0 | 3.4 (Tu) | 2.5 | 15.1 (T)  6.7 (O)  32.8 (D) | 0 | 26.1 | [156] |
| Romania (2017–2022), Disk diffusion method  N=20-137 | 5.2 (Am) 5.9 (A) | 0.7 | 8 | 3 | 42.4 (TM) | 4.7 | 23.6 (T)  24.1 (O)  16.5 (D) | 40 | 10.2 | [84] |
| Taiwan (2015-2022), Disk diffusion method N=79-94 | 65.6 (Am)  71.6 (A) | 12.8 | 17.4 | 49.5 | 19 (TM) |  | 84.4 (D)  92.6 (O) |  | 49 | [35] |
| Taiwan (2017-2022), Disk diffusion method  N=133 | 58.6 (Am)  61.7 (A) | 0.8 | 6 | 50.4 | 14 (TM) |  | 69.2 (D)  91 (O) |  | 17.3 | [49] |
| China (2021-2024), MIC (CLSI)  N=90 |  | 3.3 | 33.4 | 56.7 | 51.1 (T) | 22.3 | 93.4 (D) |  |  | [79] |
| China (2021-2023), MIC (CLSI)  N=39 | 71.8 (A) | 20.5 | 28.2 |  | 59 (TM) |  | 94.9 (T) |  | 100 | [157] |
| USA/Canada (2016-2020), MIC (CLSI)  N=250 | 9.2 (A) | 0 | 3.6 | 0 | 2 (TM)  0 (Tu) | 56.8 | 94 (T) |  | 0.8 | [80] |
| Hungary (2018-2022), MIC (CLSI)  N=164 | 36 (Am) | 0 | 52,5 | 1,2 | 4.3 (TM)  0 (Tu) |  | 55.6 (O) | 29,5 | 90,8 | [158] |
| Italy (2015-2022), MIC (EUCAST)  N=572 | 33 (A) | 10^a^ | 23^a^ | 23^a^ | 6 (Tu) |  | 53 (T) | 5 | 23 | [75] |
| Korea (2015-2025), MIC (CLSI)  N=132 | 30.3 (Am)  29.5 (A) | 2.3 | 12.9 | 50.8 | 0 (Tu)  0 (TM) |  | 83.7 (T) 90.9 (O) 16.3 (D) | 0.8 |  | [31] |
| Germany (2006-2020), MIC (CLSI)  N= 312-1647 | 5.8 (A) | 0.2 | 2.7 | 0.2 | 0 (Tu)  2.3 (TM) | 51.8 | 78.3 (T) | 2.3 |  | [71] |
| Spain (2017-2019), MIC (CLSI)  N=162 | 27.8 | 0 | 27.8 | 3 | 0 (Tu)  0.6 (TM) |  | 64.3 (D) |  | 11.1 | [159] |
| Sweden (2024-2025), MIC (CLSI)  N=74 | 0 (A9 |  | 0 | 0 |  | 12.2 | 0 (T)  0 (D) |  | 1 | [160] |
| Denmark (2024), MIC (ECOFF)  N=78 | 0 (Am) | 0 | 1.3^a^ | 0^a^ | 3.8 (Tu)  0 (TM) | 0 | 0 (D) | 0 | 0 | [161] |

Intermediate isolates are grouped with the resistant ones if information was available. CLSI cut-offs were applied wherever possible to improve comparability between studies. Data shown in this Table were calculated from the published data sets, if necessary. *Data were retrieved from recorded references and adapted by the authors to improve comparability between published studies.
